# Supplementary material for: Back-illumination interference tomography for imaging weak scattering in thick tissues
Source: Optica. 2025 Nov 20;12(12):1865–72. doi: 10.1364/OPTICA.567543 (PMC13600732; doi:10.1364/OPTICA.567543)
Supplement: Supplementary file 1 [file optica-12-12-1865-s001.pdf]

Supplemental Document

OPTICA

Back-illumination interference tomography for imaging weak scattering in thick tissues: supplement

Gregory N. McKay,[1] Jerome Mertz,[2] and Nicholas J. Durr[3,*]

[1] *Department of Surgery, The Johns Hopkins University School of Medicine, Baltimore, Maryland 21205, USA*
[2] *Department of Biomedical Engineering, Boston University, Boston, Massachusetts 02215, USA*
[3] *Department of Biomedical Engineering, Johns Hopkins University (JHU), 3400 N. Charles Street, Baltimore, Maryland 21218, USA*
[*] *ndurr@jhu.edu*

This supplement published with Optica Publishing Group on 20 November 2025 by The Authors under the terms of the Creative Commons Attribution 4.0 License in the format provided by the authors and unedited. Further distribution of this work must maintain attribution to the author(s) and the published article's title, journal citation, and DOI.

Supplement DOI: https://doi.org/10.6084/m9.figshare.30479252

Parent Article DOI: https://doi.org/10.1364/OPTICA.567543

Back-illumination interference tomography for imaging weak scattering in thick tissues: supplemental document

This document provides supplementary information about the BIT system, calculation of the coherence factor γ, and additional supplemental results.

1. CALCULATION OF THE COHERENCE FACTOR

The coherence factor (γ) according to Eq. 1, is dependent on the objective NA and the illumination NA, and plays a critical role in the generation of contrast according to Eq. 8. Since the objective NA is pre-specified at 1.15, determination of the illumination NA from $theta_{illum.}$ is all that remains. This can be accomplished using the geometry of the LED image via equation S1:

$$tan(\theta_{illum.}) = (|D_{LED}|)/\delta z' \tag{S1}$$

Where the parameters $\delta z'$ represent distance below the objective focal plane the LED is imaged, and the approximate size of the image of the LED ($|D_{LED}|$), as shown in Figure 1. These can be approximated simply using a thin-lens approach, propagating the LED a known distance away from the condenser lens (CL), where f_{CL} = 16 mm, to the objective separated by t = 115 mm, and with focal length f_{obj} = 5 mm, as outlined in S1 below.

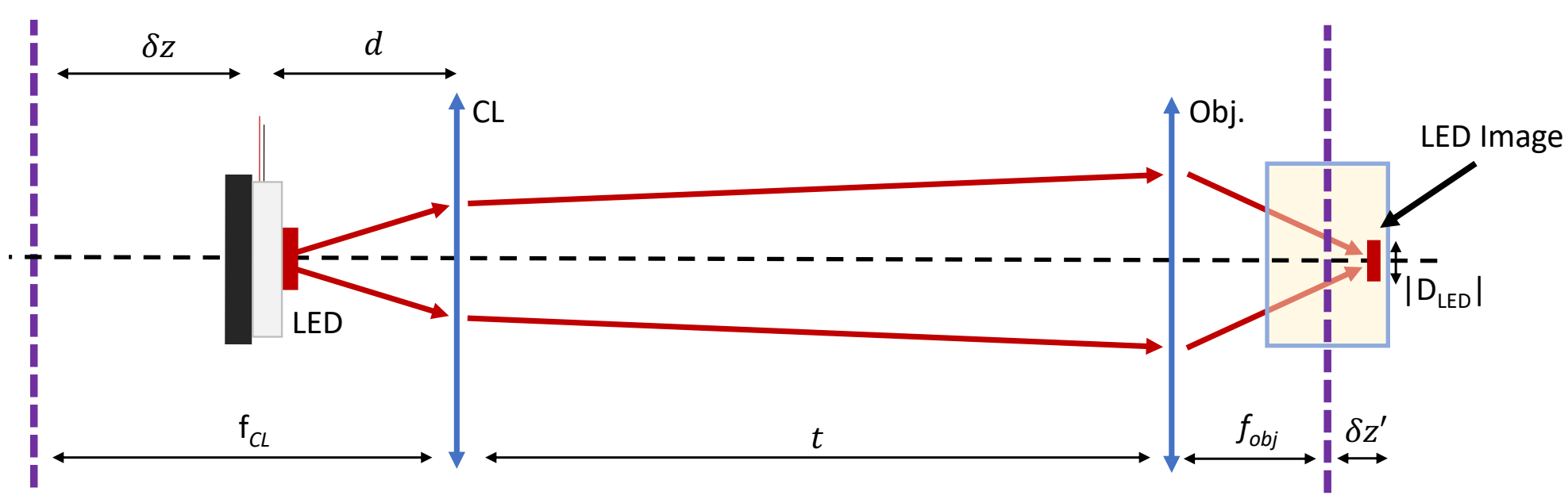

Fig. S1. A thin lens model of LED image formation within bulk scattering tissue. Note that CL represents the condenser lens, Obj. represents the microscope objective.

For varying distance of the LED from the back focal plane of CL (δz), the size of the LED image ($|D_{LED}|$), the distance beneath the focal plane ($\delta z'$), and thus the coherence factor (γ) can be computed. The results of this simulation is shown in S2. Note that these calculations are for the 530nm LED, utilized in the simulation and experiments of Figure 2, which has a physical illumination surface of 1 mm x 1 mm. Smaller sources such as the fiber illumination utilized in Figure 3, yield lower γ for a given δz.

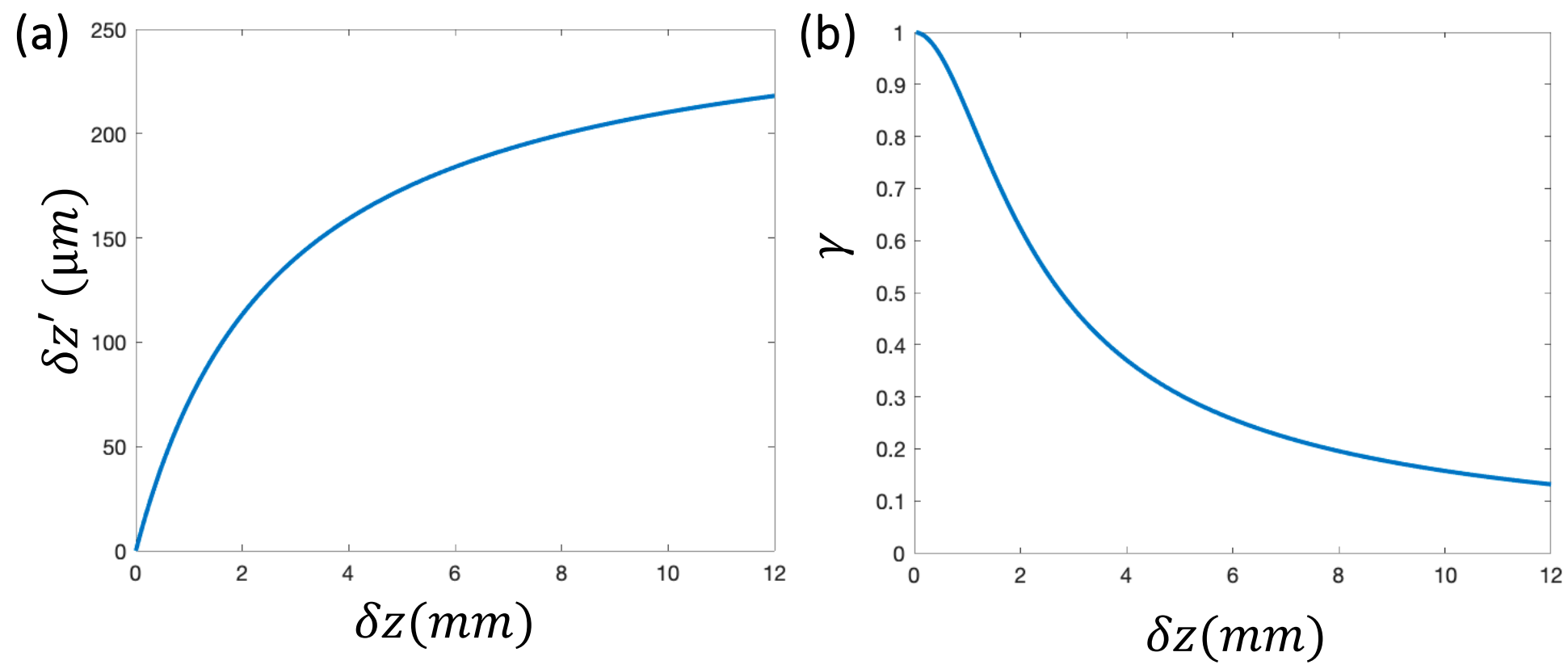

Fig. S2. (a) Conjugate relationship between δz (mm) and $\delta z'$ (μm). Note as the LED is placed closer to the condenser lens within its back focal plane, the image of the LED becomes correspondingly deeper to the objective focal plane. (b) Relationship between δz and the coherence factor γ. At $\delta z = 0$ mm, the LED is at the condensing lens back focal length and critical illumination is achieved. Coherence improves (decreasing γ) as the LED is placed closer to the condenser lens.

2. ADDITIONAL EXPERIMENTS AND DATA

To demonstrate the baseline data quality obtained with BIT, we provide example raw data from Figure 3 at varying γ values in Figure S3. Here we show Raw BIT data with increasing γ (left to right) with the full contrast range of the sCMOS [0,65536], (b) after proportional contrast adjustment with 1 % of pixels assigned below black point and 1 % of pixels above white point to improve visibility, and (c) after the flatfield correction algorithm described in the paper, whereby the original raw data is divided by a gaussian blurred version of itself to normalize intensity across the FOV.

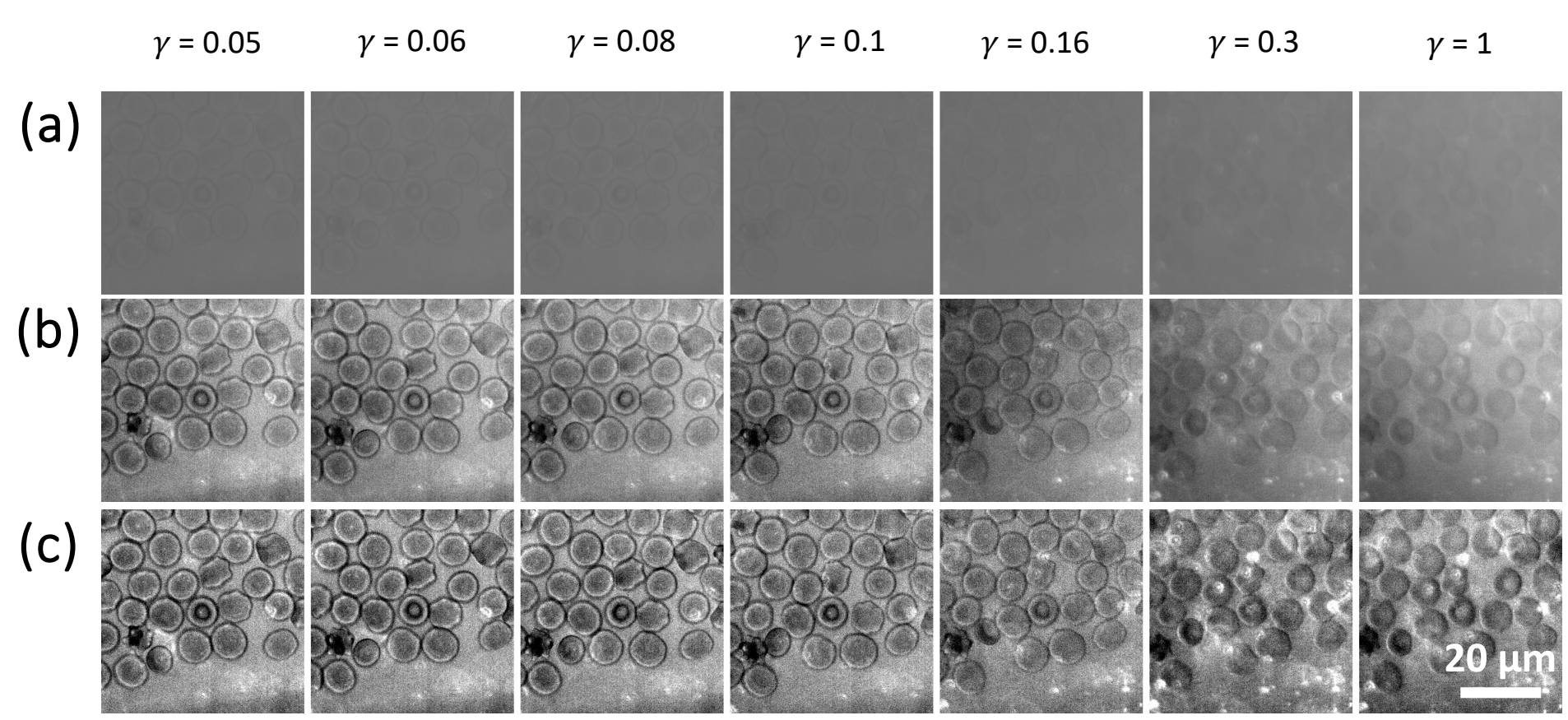

Fig. S3. (a) Raw data acquired with BIT over full contrast range of PCO.edge sCMOS [0,65536], (b) Contrast-adjusted data from (a) with 1 % of pixels assigned below black point and 1 % of pixels above white point to improve visibility, (c) Flatfield-corrected data calculated by dividing the data in (a) by a Gaussian-blurred version of itself.

In deference the work of Mazlin et. al. in their 2022 paper on Optical Transmission Tomography, we demonstrate below how BIT similarly can be used to generate tomographic slices (I_{tomo}) of scattering objects by subtracting image captured of the same object ($I(-z)$) above and below ($I(+z)$) the focal plane, via equation S2, and shown in Figure S4.

$$I_{tomo} = I(+z) - I(-z) \tag{S2}$$

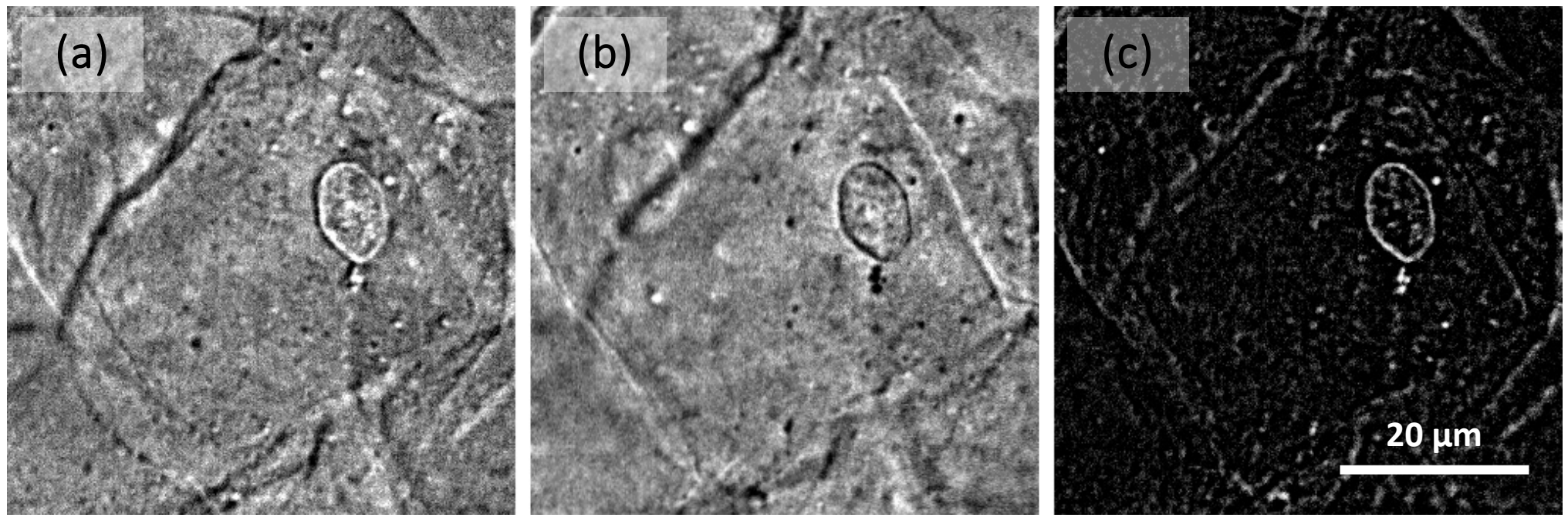

Fig. S4. (a) *In vivo* epithelial cells captured with BIT with object positioned deep to the microscope objective focal plane ($+z$), (b) the same cell imaged superficial to the microscope objective focal plane ($-z$), (c) subtraction of the two images generating a tomographic slice.

Lastly, to demonstrate the ability of BIT to image into tissue depth, we present axial slices acquired in bulk *ex vivo* human duodenum acquired with BIT at 660 nm in Figure S5. BIT contrast is clearly visible at least 50 μm into the sample. Future work will focus on further characterizing BIT imaging in different tissue types and creating 3D reconstructions using the tomographic demonstrated in Figure S4.

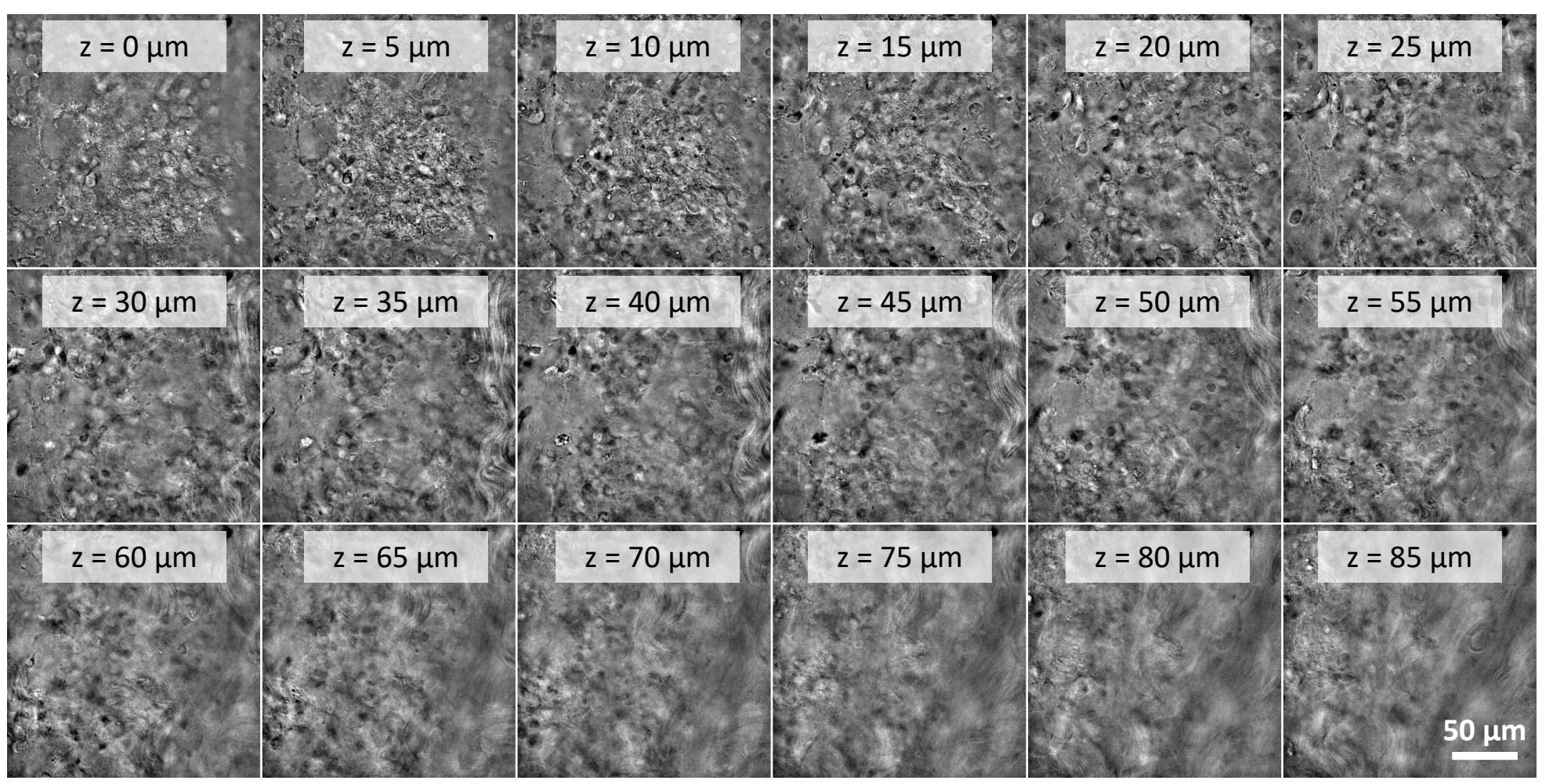

Fig. S5. *Ex vivo* bulk tissue imaging of human duodenum with BIT demonstrating contrast at least 50 μm into the sample.
